# Recurrent Structural Motifs in Non-Homologous Protein Structures

**DOI:** 10.3390/ijms14047795

**Published:** 2013-04-10

**Authors:** Maria U. Johansson, Vincent Zoete, Nicolas Guex

**Affiliations:** 1 Vital-IT Group, SIB Swiss Institute of Bioinformatics, CH-1015 Lausanne, Switzerland; 2 Molecular Modelling Group, SIB Swiss Institute of Bioinformatics, CH-1015 Lausanne, Switzerland; E-Mail: vincent.zoete@unil.ch

**Keywords:** Delaunay triangulation, protein fragments, long-range contacts, protein folding, protein structure, structural motifs, structure comparison/similarity, structure prediction

## Abstract

We have extracted an extensive collection of recurrent structural motifs (RSMs), which consist of sequentially non-contiguous structural motifs (4–6 residues), each of which appears with very similar conformation in three or more mutually unrelated protein structures. We find that the proteins in our set are covered to a substantial extent by the recurrent non-contiguous structural motifs, especially the helix and strand regions. Computational alanine scanning calculations indicate that the average folding free energy changes upon alanine mutation for most types of non-alanine residues are higher for amino acids that are present in recurrent structural motifs than for amino acids that are not. The non-alanine amino acids that are most common in the recurrent structural motifs, *i.e.*, phenylalanine, isoleucine, leucine, valine and tyrosine and the less abundant methionine and tryptophan, have the largest folding free energy changes. This indicates that the recurrent structural motifs, as we define them, describe recurrent structural patterns that are important for protein stability. In view of their properties, such structural motifs are potentially useful for inter-residue contact prediction and protein structure refinement.

## 1. Introduction

The sequence of a protein largely determines its fold, but the folding process cannot realistically be achieved through an exhaustive exploration of the conformational space, and it is believed that proteins fold through a combination of local structural arrangements, such as helices and long range interactions that will lead to the formation of compact hydrophobic cores and/or sheets. Protein structures as we observe them in experimentally determined structures reflect the global energy minimum for a given protein. Structural genomics initiatives (e.g., Montelione *et al.*[1]) have made considerable progress in rapid large-scale determination of protein structures by X-ray crystallography and nuclear magnetic resonance (NMR) spectroscopy. The main goal of these initiatives has been to systematically target many large families without structural coverage, as well as very large families with inadequate structural coverage. As a result, this has both improved our structural leverage [2] and shown that it has been increasingly difficult to discover previously unobserved protein structures (e.g., folds or fragments [3]). However, the ratio of known experimental protein structures to the number of all sequenced proteins is still less than one in 1,000 [4] and is expected to continue shrinking. Given the wealth of structural data accumulated to date, the protein structure prediction challenge still consists in the identification of the best template/fragments/parts to combine to accurately predict a given protein structure. For the foreseeable future, there will still be a demand for efficient and accurate prediction of protein three-dimensional (3D) structures and the improvement of existing methods, as well as the development of new approaches which will be necessary [5].

In the protein structure prediction and protein modeling setting, Bowie and Eisenberg [6] pioneered the systematic use of sequentially contiguous fragments extracted from known experimentally determined structures to assemble new structures. In concept, this approach is closely related to the use of fragments of known structures to speed up experimental structure determination through automated fitting of measurement data to structure fragments [7–11], but in the former case, no experimental data is available to guide the choice between different fragment candidates. Over time, a variety of protein fragment collections with fixed or variable lengths have been assembled, such as the ones described in [12–17].

The approach of forming complete structures by assembling small fragments from different experimentally determined structures has subsequently been developed further and constitutes an essential component of currently successful and generally applicable *ab initio* protein structure prediction methods, such as Rosetta [18–21], Fragfold [22], Simfold [23] and TASSER [24–27]. Rosetta, for example, operates by combining small sequential fragments (three and nine residues long) to build a framework on which side chains are reconstructed using rotamer libraries with idealized bond lengths and angles, followed by an all-atom coordinate optimization using a classical molecular mechanics force field as the final step of the method.

Catalogues of fragments of recurring sequence-structure patterns have already proven themselves useful when identifying catalytic sites [28,29], predicting coiled-coils [30] and detecting different kinds of structural analogs [12] and structural neighbors [31] and are expected to be useful also for structure prediction and experimental structure determination, as mentioned above. However, most catalogues of such patterns essentially only consider sequentially contiguous stretches of amino acid residues, and to the best of our knowledge, there is at present no comprehensive catalogue of sequentially non-contiguous structural motifs that occur repeatedly in unrelated structures.

In this paper, we present an extensive collection of recurring structural arrangements of 4–6 sequentially non-contiguous amino acid residues extracted from 5489 distinct Protein Data Bank (PDB) chains with at most 20% sequence identity. Our objective with this collection of structural patterns is not to represent the most probable conformation for a given sequentially non-contiguous set of residue types, but to capture the entire distribution of conformations that selected combinations of residue types and residue sequence separations are likely to adopt in protein structures, given that the residues in question are in close spatial proximity.

Recurring patterns of structural arrangements of sequentially non-contiguous amino acids do not only represent frequently occurring and, thereby, statistically more likely inter-residue contacts, making them potentially useful for long-range contact-prediction, they have also previously demonstrated themselves to be indicative of properties, such as binding affinity [32,33], catalytic activity [34,35], as well as intrinsic stability, as we have recently pointed out elsewhere [36]. The present collection of such patterns could therefore prove to be a useful resource for protein structure modeling, protein structure refinement and a complement to the current flora of protein structure databases, which focus on sequentially contiguous fragments.

## 2. Results and Discussion

### 2.1. Protein Sets

**Figure 1:**
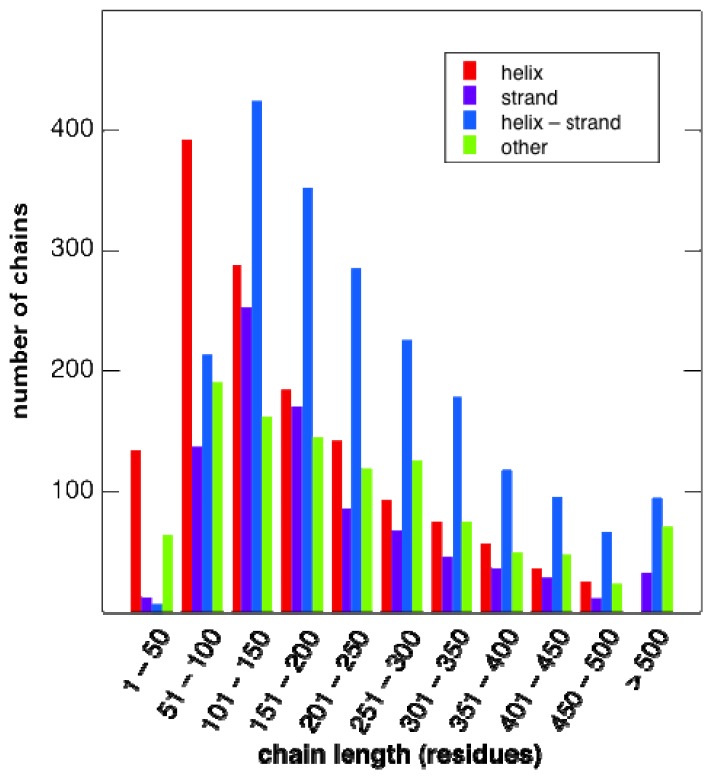
Protein length distributions. The number of protein chains of different lengths in the motif set, classified by secondary structure content, as described in Section 3.2.

A set of 31,980 protein chains with a maximum pairwise sequence identity of 99% was selected as described in Section 3.1 and is referred to as the search set. From the search set, a subset of 5489 chains, referred to as the motif set, was selected, such that no chain in the latter set has more than 20% sequence identity with any other chain in it (see Section 3.1 for details). This of course does not preclude the presence of evolutionarily-related proteins in our motif set (see [37] for an interesting discussion about evolution with respect to folds). However, given the short discontinuous size of our motifs (4–6 residues) and the low overall sequence identity ensured between members of our motif set, we felt that any recurrent instances of strict conservation of our 4–6 residue motifs in other proteins should have interesting properties with respect to their intrinsic compactness/stability. Furthermore, given the nature of our motifs, a precise multi-level fold classification, such as Structural Classification of Proteins (SCOP) [38] or CATH [39], was not required, and to report our results, protein chains were instead classified as belonging to exactly one of the four categories: helix, strand, helix-strand mixture and “other”, depending on the fraction of residues in the corresponding secondary structure conformation in each protein chain, as described in Section 3.2. Given the mentioned classification scheme, the motif set contains 1483 helix protein chains, 877 strand protein chains, 2061 helix-strand mixture protein chains and 1068 other protein chains. In the present study, we have not distinguished between soluble and membrane proteins. The empirical chain-length distribution of protein chains in the motif set is shown for each of the four classes of protein chains in Figure 1.

### 2.2. Identification of Non-Contiguous Recurrent Structural Motifs

**Figure 2:**
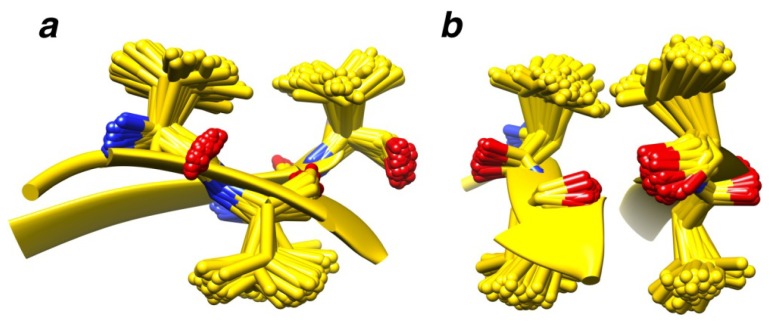
A very commonly occurring RSM. Structural motifs seen (**a**) from the side and (**b**) rotated 90 degrees about the *y*-axis. The 2 + 2 RSM with the highest level of recurrence is shown. It is found in 108 mutually unrelated chains.

Geometric atom configurations are considered to be replicated when the coordinate root mean square distance (RMSD) similarity measure falls below predetermined cutoff values. For each chain in the motif set, we extracted candidate structural motifs composed of two groups of residues, referred to as pieces of the structural motif, separated along the peptide chain by at least six residues (see Figure 11). Then, all geometric backbone configurations of 4–6 amino acid residues with sequence spacing properties, as described in Section 3.3, and that repeat themselves in at least two other mutually unrelated chains (BLAST [40,41]*E*-value > 10.0) from the search set, were identified. The different kinds of candidate structural motifs and recurrent structural motifs are denoted by the number of residues in their two pieces, separated by a plus sign (e.g., 2 + 2, 2 + 3, *etc.*). Thus, amino acid backbone packing patterns that are present in three or more mutually unrelated (BLAST *E*-value > 10.0) search set chains are identified and categorized as *M + N* recurrent structural motifs (RSMs). The total number of such *M + N* amino acid residue geometric backbone configurations that were identified as repeating themselves and, thereby, defining RSMs are shown in Table 1, along with the corresponding average degree of RSM recurrence observed for different similarity measure cutoff values. It can be seen that our RSMs are clearly composed mostly of amino acids from secondary structure elements. For example, a very commonly occurring RSM is shown in Figure 2. It consists of two pairs of valines and is found in 108 mutually unrelated chains. The RSM shown in Figure 2 is the one with the highest value of recurrence among all our RSMs. This motif is found in chains classified into 36 different SCOP families (and 33 superfamilies, 32 folds and four classes). However, only 33% of the supporting chains have a SCOP classification, but it is nonetheless clear that it is a very widely spread structural motif. The twist of the beta-sheet in Figure 2 is caused by the interactions between the side chains of the valines; this was first described by Chou and Scheraga [42].

**Figure 3:**
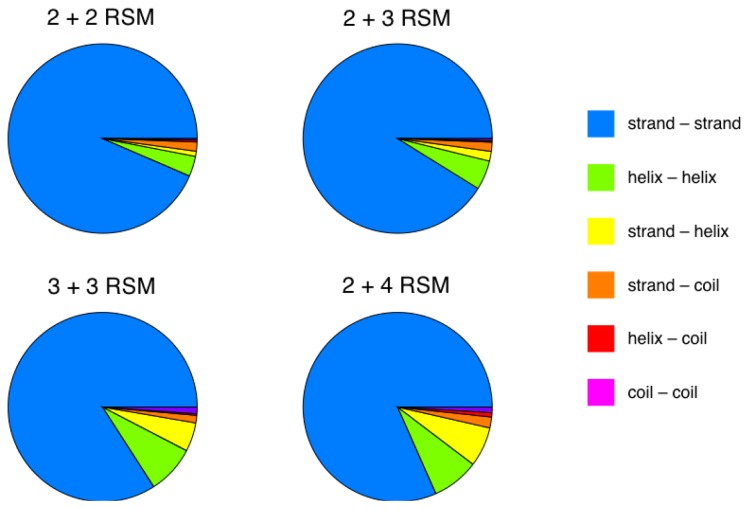
Relative frequencies of RSMs with residues in their two pieces (see Section 3.3) adopting various combinations of secondary structure, for different sized RSMs, at the lowest RMSD cutoff value for each RSM-type (see Table 1).

The requirement, as mentioned above, that all counted instances of each RSM must derive from very distant protein sequences is imposed in order to only retain structural similarities that are due to inherent structural and folding constraints. We investigated the relative proportions of various secondary structure types present in the RSMs obtained in more detail (Figure 3). The strand-strand RSM-types are by far the most common (>80%). The helix-helix RSM types are the second most common, and their relative proportion increases with the size of the motif. The overall proportion of strand-strand RSMs decreases with increasing RMSD cutoff values and the overall proportion of helix-helix RSMs increases with increasing RMSD cutoff values (data not shown). For example, for 2 + 2 RSMs, the helix-helix proportion increases from 3.5% (0.4 Å) to 8.8% (0.8 Å), and for the 2 + 3 RSMs, the helix-helix proportion increases from 5.0% (0.5 Å) to 7.3% (0.9 Å). This indicates that the low proportion of helix-helix RSMs arises because helix-helix contacts are more sensitive to RMSD cutoffs for the ranges of such values used here, possibly accommodating a broader range of relative orientations than strands, rather than it being a consequence of an unsuitably chosen motif set and/or motif set size (see Section 2.1 for details about how the motif set was chosen).

The values in parentheses in Table 1 show the number of unique RSMs, when RSMs are not distinguished using geometric properties (*i.e.*, considering only residue-type, sequence separation and secondary structure characteristics). These values represent lower bound estimates on the number of geometrically distinct RSMs of each corresponding type for different similarity measure cutoff values.

### 2.3. Amino Acid Composition of RSMs

**Figure 4:**
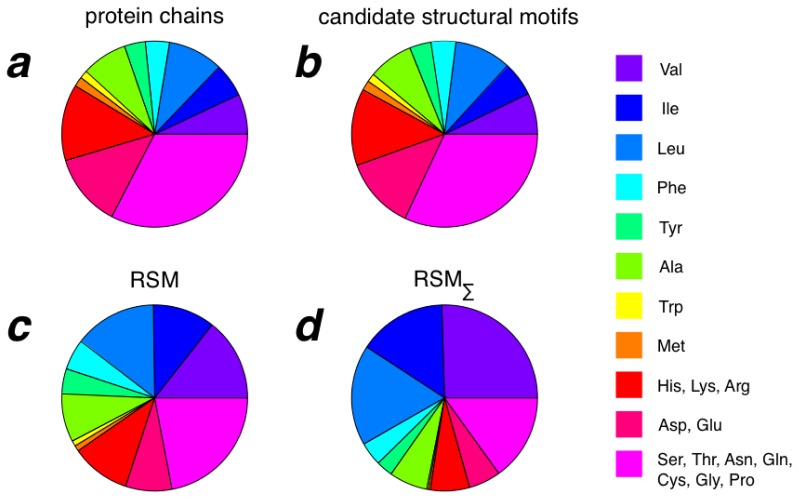
Amino acid compositions as seen over all residues in (**a**) all chains, (**b**) all candidate structural motifs and (**c**) all RSMs. In the case of (**d**), amino acid composition has been summed overall RSMs, counting each residue as many times as it is present in RSMs.

When calculating the amino acid composition of complete protein chains, of candidate structural motifs and of RSMs, no substantial difference is found between complete protein chains and candidate structural motifs (Figure 4a,b). For RSMs, on the other hand, Val, Ile, Leu, Phe and Tyr are relatively more abundant than in both complete chains and candidate structural motifs, whereas the objectively most abundant residue types in RSMs are Val, Leu, Ile and Ala (Figure 4c). That Val, Ile and Leu are found in many RSMs is evident from Figure 4d, in which amino acid compositions have been summed over all RSMs without compensation for the fact that residues present in multiple RSMs are then counted multiple times. Thus, RSMs appear to be built up from residues commonly found in protein cores, and we therefore expect them to be useful for protein structure prediction and refinement. However, evaluation of the usefulness of RSMs for structure prediction and refinement is beyond the scope of the present paper. The individual values for all amino acid types can be found in Table S1 in Supplementary Information. From Table S1, for example, it is clear that there is no increased Cys presence in RSMs compared to protein chains and candidate structural motifs, which exclude a major contribution of disulfide bridges in our RSMs.

### 2.4. Coverage

When determining the degree of recurrence of each candidate structural motif, mutually related repeated occurrences have been carefully excluded, and each candidate structural motif that occurs at least three times in mutually unrelated chains is accepted as an RSM. The residues that are present in a RSM defined from a given chain are said to be covered by a RSM. According to our definition, a RSM is a candidate structural motif such that sufficiently similar instances (of the RSM) have been found in at least two other mutually unrelated chains from the search set. These instances are said to support the coverage of each residue covered by that RSM.

**Figure 5:**
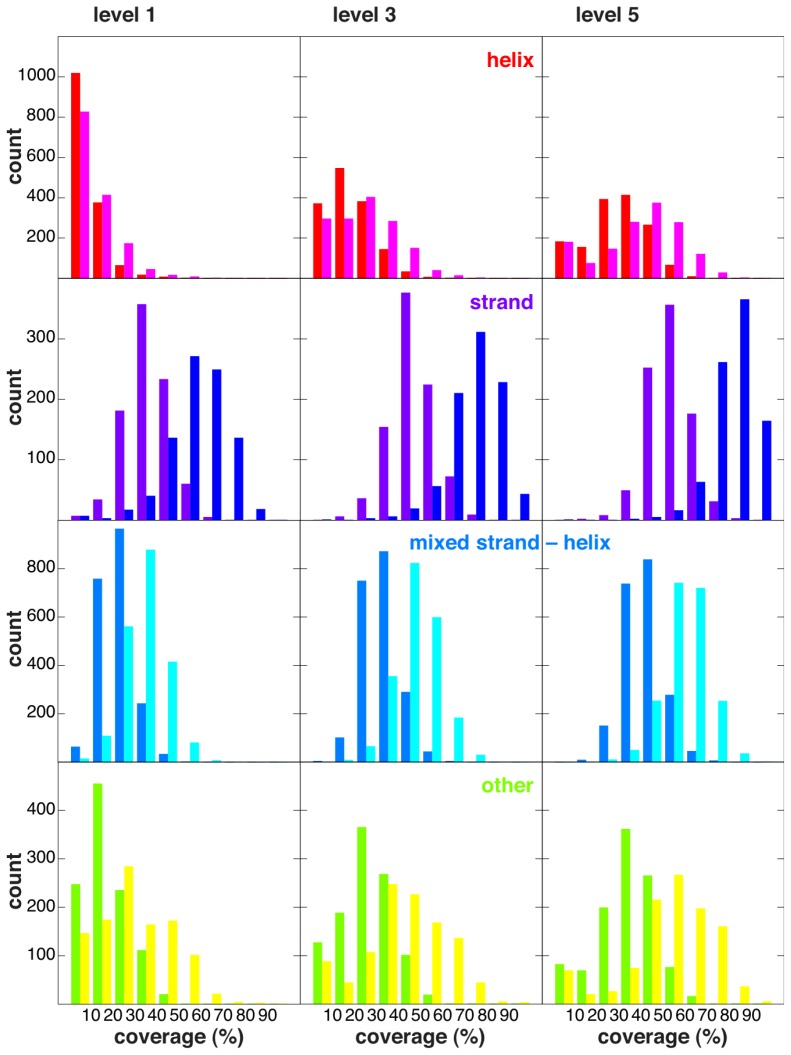
Chain coverage distributions. Coverage distribution for chains of different secondary structure classification with respect to all residues/helix and strand residues only, at different RMSD cutoff values (level 1, level 3 and level 5 from left to right, as defined in Table 1) of each RSM type. For helix-class chains, the coverage is shown for all residues (in red) and for helix and strand residues only (in magenta). For strand-class chains, the coverage is shown for all residues (in purple) and for helix and strand residues only (in dark blue). For mixed strand-helix-class chains, the coverage is shown for all residues (in blue) and for helix and strand residues only (in cyan). Finally, for other-class chains, the coverage is shown for all residues (in green) and for helix and strand residues only (in yellow).

The coverage distributions of the 5489 chains of our motif set are shown in Figure 5, for the four different secondary structure classes as defined in Section 3.2. On average, it is clear that strand-class protein chains are covered to a substantially higher degree than helix-class protein chains and that protein chains classified as mixed helix-strand or other are typically covered to degrees falling between the extremes represented by helix- and strand-class protein chains. However, quite a few helix-class chains consist of a single helix, and in such cases, it is not expected to obtain a large number of RSMs. Figure 5 also presents the coverage distributions that arise when only residues in helix or strand conformations are considered to be coverable, and since most RSMs only cover helix and/or strand residues (see Table 1 and Figure 3), coverage distributions clearly shift toward higher values in this case.

**Figure 6:**
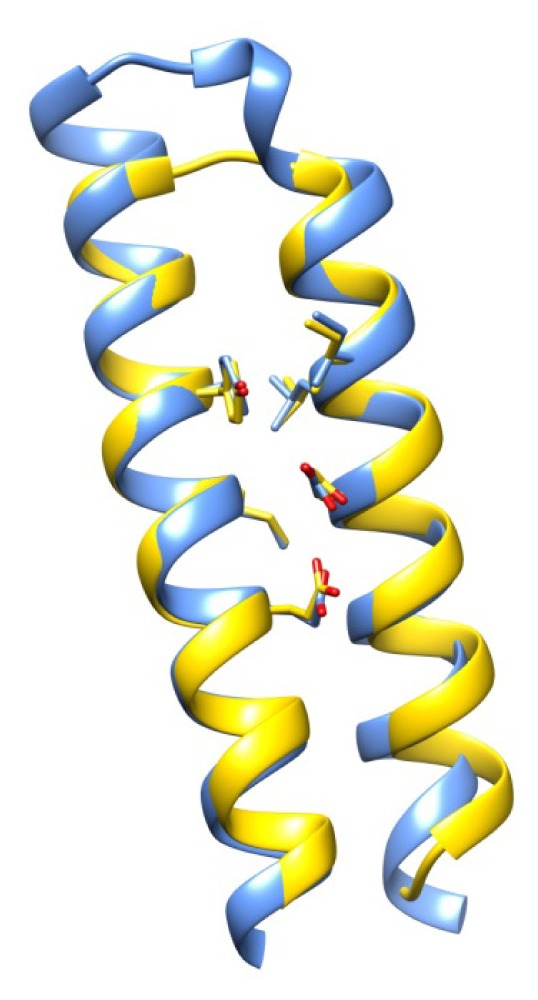
The protein chain with the largest fraction of covered residues, 1jm0-A (yellow), supported by a common chain, 3ogh-A (blue). The residues covered by RSMs stabilize the central part of the helix-helix packing.

If relatedness has been successfully eliminated from configurations of coverage-supporting chains, it is expected that the coverage of most motif set chains is supported by a heterogeneous collection of search set chains and, in particular, that coverage-supporting chains only support the coverage of a relatively small number of residues of a given motif set chain. The protein chain with the largest number of covered residues supported by a common chain is 3rgz-A, for which 114 out of 745 theoretically coverable residues are supported by the chain, 3o6n-A. Both proteins adopt a similar fold, although they share only about 20% identity in a common region, where multiple indels are also found. This is an extreme example, and the protein chain with the second largest number of residues supported by a common chain is 2o6s-A, for which 49 out of 208 theoretically coverable residues are supported by a common chain. Correspondingly, the protein chain with the largest fraction of covered residues supported by a common chain is 1jm0-A, which contains two antiparallel helices of a synthetic *de novo* chemically synthesized four helix bundle protein [43]. For 1jm0-A, 29% out of 48 theoretically coverable residues are supported by the chain, 3ogh-A, which is also a four helix bundle. Noticeably, these two chains present only about 23% identity once ideally superposed, and the residues covered by the RSMs stabilize the central part of the helix-helix packing (Figure 6). The just presented values for coverage support represent extreme values, however, and the average number and fraction of covered residues supported by a common chain is only 8.5% ± 5.1% and 4.8% ± 3.2%, respectively, which is compatible with the expectation that chains typically do not support a large number/fraction of covered residues in any chain and, thus, that the coverage values reported in Figure 5 are not due to a small number of chains that provide widespread and extensive coverage support.

### 2.5. More Compact Candidate Structural Motifs Lead to Relatively More RSMs and Higher Recurrence

One collection of RSMs was calculated while imposing an upper distance limit of 10 Å on non-local (see Section 3.3) inter-residue contacts, as represented by edges in Delaunay triangulations, and another collection of RSMs was calculated without imposing any such distance limits. The former collection only contains 2% fewer RSMs than the latter, even though 25% of all candidate structural motifs were eliminated by imposing the 10 Å upper distance limit, suggesting that non-compact candidate structural motifs are less likely to lead to RSMs. Such upper distance limits on inter-residue contacts have previously been used in other studies [44], and an upper distance limit of 10 Å on contacts was imposed for all RSMs that we present (e.g., see Table 1).

A further increase of the compactness requirements, on the other hand, obtained by requiring that every residue in an RSM must be in contact with all residues in the other piece (see Section 3.3) of that RSM, leads to rejection of large numbers of candidate structural motifs and, subsequently, to low levels of coverage, especially for the larger size RSMs. Indeed, with growing numbers of residues in RSMs, it rapidly becomes structurally impossible for each residue to be in direct contact with every residue in all other pieces of the RSM. At the lowest RMSD cutoff values (see Table 1), we have identified a total of 531,472 RSMs, but by requiring that each residue in each RSM be in contact with all residues in other pieces of the RSM, only 135,582 RSMs are found. Thus, almost 400,000 RSMs are lost (losses are 71% for size 4 RSMs, 87% for size 5 RSMs 93% for 2 + 4 RSMs and 100% for 3 + 3 RSMs). Ignoring size 6 RSMs (since they are too few to draw conclusions from), the average recurrence when requiring that all residues are in contact with each other are: 7.2, 5.3, 3.8 and 3.0, for size 4 RSMs containing only helices or strands, size 4 RSMs containing coils, size 5 RSMs containing only helices or strands and size 5 RSMs containing coils, respectively. These values are for the most part slightly higher and never lower than the corresponding values given Table 1, which were obtained without imposing the denser inter-residue contact requirements that we have just described. However, as already mentioned, the slight increase in recurrence carries a high price in terms of reduced coverage and total number of RSMs.

**Figure 7:**
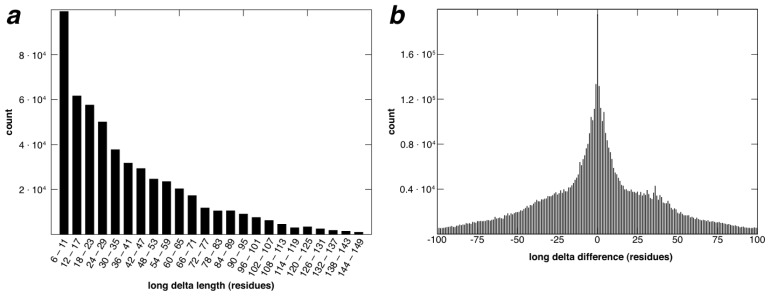
Long delta properties. (**a**) Distribution of long delta lengths for the RSMs at the lowest cutoff value in Table 1; (**b**) distribution of differences between long delta values for RSMs in Table 1 (lowest RMSD cutoff) and their associated search-set instances.

In Figure 7, the distribution of long delta lengths (e.g., the number of residues that separate the two pieces, *P*_1_ and *P*_2_, of the *M + N* RSMs) and the distribution of differences between long delta values of the motif set proteins and their associated search-set instances are shown. In (a), 50% of all long delta values are ≤29, 90% are ≤85, 95% are ≤104 and 99% ≤150. Similarly, in (b), 50% of the absolute values of all long delta differences are ≤20, 90% are ≤66, 95% are ≤80 and 99% ≤96. In calculating differences in long delta values between RSMs and the corresponding residues in supporting chains, each unique long delta value is counted at most once for each group of mutually related supporting chains.

### 2.6. Computational Alanine Scanning

**Figure 8:**
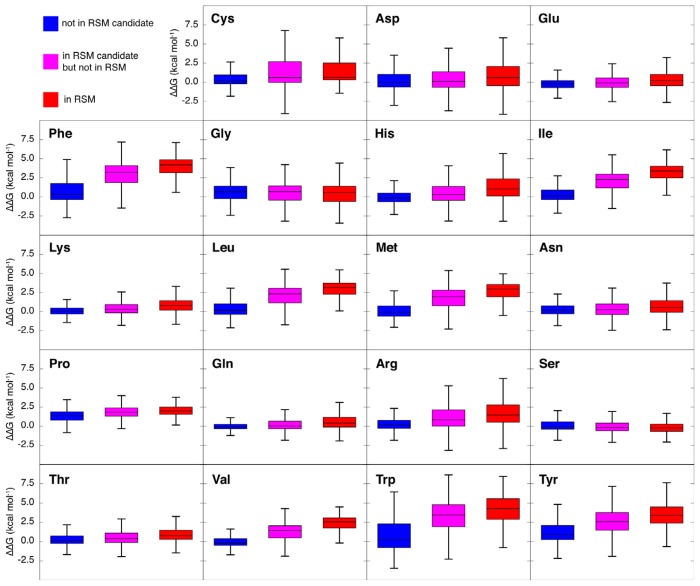
Folding free energies (kcal mol^−1^) from computational alanine scanning calculations, shown for each kind of non-Ala residue. Values in red are those for residues that take part in RSMs. Values in magenta are those for residues that were considered in RSM candidates, but that turned out to be non-recurrent. Values in blue are for residues that are never part of any RSM candidate. The tops and bottoms of the boxes indicate the upper and lower quartiles (*i.e.*, *Q*3 and *Q*1, respectively) of each corresponding sample, and the whiskers indicate the corresponding highest and lowest value inside the bound given by *Q*3 + 1.5 · *IQR* and *Q*1–1.5 · *IQR*, respectively (where *IQR* denotes the so-called interquartile range, *Q*3–*Q*1). The line within each corresponding box indicates the median of each sample.

Computational alanine scanning calculations indicate that the average folding free energy changes upon alanine mutation for most types of amino acids are higher if the amino acid is covered by one or more RSMs than if it has never been part of a single non-contiguous candidate structural motif (Figure 8). This is especially the case for Phe, Ile, Leu, Met, Val, Trp and Tyr, for which the average ΔΔG is significantly (*p* < 0.001, as determined by a Wilcoxon rank-test) greater than 2 kcal mol^−1^ in RSMs (red in Figure 8) and at least 2 kcal mol^−1^ significantly larger (*p* < 0.001) than their corresponding values when not in candidate structural motifs (blue in Figure 8). This suggests that the RSMs, as we define them, describe recurrent packing patterns that are important for protein stability. In Figure 8, we also show in magenta the folding free energy changes for residues that take part in candidate structural motifs, but do not lead to RSMs (*i.e.*, do not appear in three or more mutually unrelated protein structures). They tend to have intermediary average ΔΔG.

### 2.7. Examples

To check that our RSMs are indeed able to capture recurrent arrangements of conformations that could be applied in the future in the context of protein structure prediction, we have examined 2dbs-A and 2in5-A in more detail. Both structures are structural genomics consortia targets and have unknown/hypothetical functions. They do not have any sequence similarity to any protein in the RCSB protein data bank and have at most one structure in the PDB identified as being structurally similar [45]. For 2dbs-A, the similar structure is 1a79 (max sequence identity: 8%, RMSD 3.16 Å) and for 2in5-A, the similar structure is 3fzx (max sequence identity: 9%, RMSD 3.29 Å). Both 2dbs-A and 2in5-A belong to the New Fold category in SCOP.

**Figure 9:**
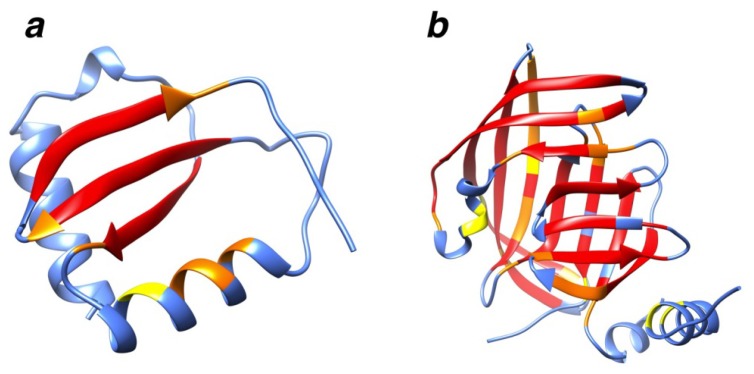
(**a**) Extent of coverage for 2dbs-A. In total, 17 residues of 2dbs-A (80 residues) are covered by 35 RSMs, leading to a coverage of 21.3% for the complete chain at backbone RMSD level 1 (red). At higher backbone RMSD levels, more RSMs cover 2dbs-A. At level 3, seven more residues are covered (orange), and at level 5, one additional residue is covered (yellow). The coverage increases to 30.0% and 31.3% at levels 3 and 5, respectively; (**b**) Extent of coverage for 2in5-A. In total, 81 residues of 2in5-A (195 residues) are covered by 82 RSMs, leading to a coverage of 41.5% for the complete chain at backbone RMSD level 1 (red). At higher backbone RMSD levels, more RSMs cover 2in5-A. At level 3, 22 more residues are covered (orange), and at level 5, seven additional residues are covered (yellow). The coverage increases to 52.8% and 56.4% at levels 3 and 5, respectively.

Both structures are covered by RSMs (Figure 9). In total, 2dbs-A is covered by 35 RSMs and 2in5-A is covered by 82 RSMs. The coverage of 2dbs-A is supported by chains belonging to 42 different SCOP families (and 33 superfamilies, 30 folds and four classes) and most of the supporting chains belong to the SCOP family c.82.1.1. The coverage of 2in5-A is supported by chains belonging to 67 different SCOP families (and 58 superfamilies, 53 folds and five classes), and of these SCOP families, most of the supporting chains belong to either b.42.1.1 or d.22.1.1. Furthermore, far from all chains in the search set have been classified in SCOP. For 2dbs-A and 2in5-A only 34% and 38%, respectively, of the supporting chains have a SCOP classification.

### 2.8. Side Chain Packing

**Figure 10:**
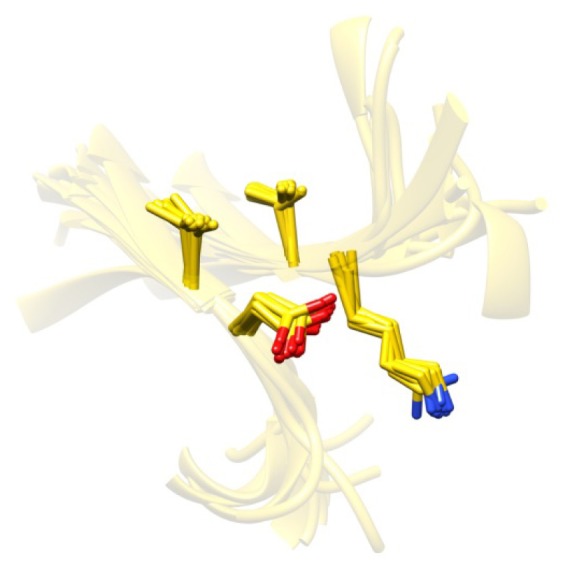
An RSM with identical side chain packing. The 2 + 2 RSM with the highest level of recurrence is shown. It is found in 15 mutually unrelated chains. The side chain of the Asp seems to hydrogen bond to the main chain of the Lys in the other strand.

The RSMs presented so far have only been required to satisfy backbone similarity requirements, and they thus allow for some leeway in side chain conformations. We expect them to capture the full distribution of corresponding residue type and sequence separation combinations and, thereby, lead to more realistic refinement constraints. In Table 2, we report the number of RSMs found and their average degrees of recurrence when using similarity measures that involve all heavy atoms of amino acid residues and not only backbone atoms, as shown in Table 1. Imposing stringent RMSD cutoffs for all heavy atoms instead of only for backbone atoms leads to noticeably fewer RSMs, and RSMs satisfying such requirements will most likely not accurately capture the full distribution of possible side chain conformations. Nonetheless, such RSMs are interesting in themselves, as they identify residue combinations and backbone geometries that tend to maintain identical side chain packings (e.g., see Figure 10) and could have uses in the engineering of stable proteins, for example. Note, however, that no size 6 RSMs of either kind were identified, and the corresponding columns are therefore not present in Table 2.

## 3. Experimental Section

### 3.1. Selection of Protein Sets

The PISCES protein sequence culling server [46] (settings: resolution 3.0 Å or better, sequence length 40–10,000 and ignoring non-X-ray and C^α^-only entries) was used to create a subset of all protein chains in the RCSB Protein Data Bank [47] (http://www.RCSB.org) on November 2011, such that the maximum pairwise sequence identity between chains in the subset is 99%. This resulted in a subset of the PDB containing 31,980 protein chains. Hereafter, this set, essentially representing all structures present in the PDB, is referred to as the search set. From the search set, a reduced collection of 5493 PDB chains having a maximum sequence identity of 20% was extracted (again, using PISCES and with the settings mentioned above). Four PDB chains were removed from the collection of 5,493 chains, because they did not have a complete RCSB BLAST table (described in more detail below). The remaining 5489 PDB chains are referred to as the motif set, from which the candidate structural motifs have been generated.

### 3.2. Protein Chain Classification

Protein chains with 40% or more residues in helix conformation and less than 15% of their residues in strand conformation are classified as being helix chains. Protein chains with 40% or more residues in strand conformation and less than 15% of their residues in helix conformation are classified as being strand chains. Protein chains with more than 20% of their residues in helix conformation and more than 20% of their residues in strand conformation are classified as mixed helix-strand chains. Finally, all protein chains not falling into any of the three mentioned classes are categorized as “other”.

### 3.3. Sequence Properties of Non-Contiguous Candidate Structural Motifs

Swiss-PDBViewer has recently been augmented with the ability to search for structural motifs in large collections of protein structures [36] and is available for general public use via http://spdbv.vital-it.ch/. Briefly explained, a so-called motif specification containing constraints on various aspects of a motif must be provided, and the search machinery then locates and reports all-atom conformations that satisfy the provided constraints. In a motif specification, it is possible to state constraints on acceptable amino acid residue-types, acceptable secondary structure (helix, strand, coil or any combination thereof), acceptable distances between specific pairs of atoms and acceptable sequence separation between amino acid residues. We have also developed programs external to Swiss-PDBViewer, whereby motif specifications (as detailed below) can be automatically generated. These pieces of software are the main tools with which we have generated the collections of non-contiguous recurrent candidate structural motifs described here.

**Figure 11:**
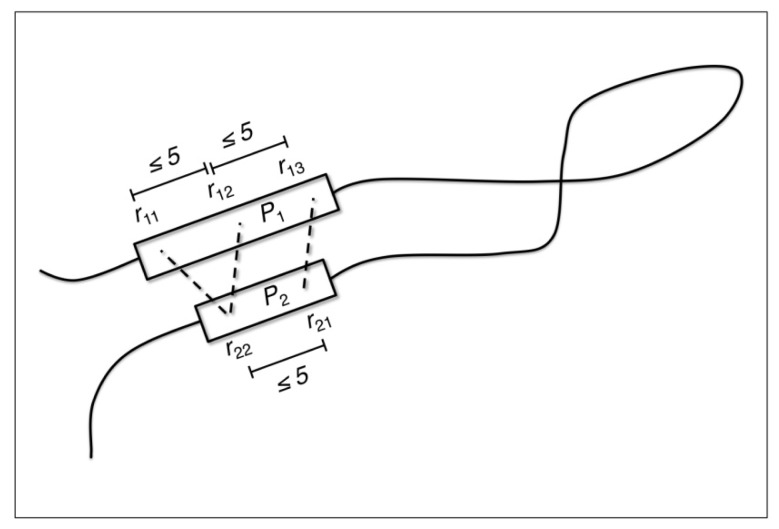
Conceptual motif definition. A graphical illustration of how residues *r*_1,1_,...,*r*_1,3_ and *r*_2,1_,...,*r*_2,2_, constituting pieces *P*_1_ and *P*_2_, respectively, combine to form a 2 + 3 candidate structural motif. The two pieces *P*_1_ and *P*_2_ must be at least 6 residues apart along the protein chain (or alternatively expressed, *r*_21_ − *r*_13_ ≥ 6).

The main features of our principal set of candidate structural motifs are that they are composed of two sequentially non-adjacent pieces of amino acid residues, *P*_1_ = (*r*_1,1_,…,*r*_1,_*_M_*) and *P*_2_ = (*r*_2,1_,…,*r*_2,_*_N_*), such that each residue is one of the 20 standard amino acids and that its atom-coordinates are defined by PDB-format ATOM records (*i.e.*, residues with one or more atom coordinates given by HETATM records are not present in candidate structural motifs, even if these residues are one of the 20 standard amino acids). As also depicted in Figure 11, each piece of amino acid residues, *P*_1_ and *P*_2_, comprises two or three residues (*i.e*., 2 ≤ *M* ≤ 3 and 2 ≤ *N* ≤ 3), such that seqno(*r*_1_,*_i +_*_1_) − seqno(*r*_1_*_,i_*) ≤ 5 for 1 ≤ *i* ≤ *M* − 1 and seqno(*r*_2_,*_j +_*_1_) − seqno(*r*_2_*_,j_*) ≤ 5 for 1 ≤ *j* ≤ *N −* 1, where seqno(*r*) denotes the sequence number of residue *r* and such that all residues in the range *r*_1,1_,…,*r*_1_*_,M_* have consistently been assigned one of either helix or strand secondary structure and all residues in the range *r*_2,1_,…,*r*_2_*_,N_* have also consistently been assigned one of either helix or strand secondary structure (in both cases, according to the secondary structure definitions used by Swiss-PDBViewer [48,49]). In addition, the two pieces of each candidate structural motif must have a sequence separation of at least six residues (*i.e.*, seqno(*r*_2_,_1_) − seqno(*r*_1_*_,M_*) ≥ 5). During the search for RSMs, residue sequence separations are seen as being constant within each piece, *P*_1_ and *P*_2_, of a candidate structural motif (e.g., the structural motifs found in the search set must present the same sequence separation as the candidate structural motif defined in the motif set). However, the sequence separation between pieces *P*_1_ and *P*_2_ is permitted to vary (e.g., the structural motifs found in the search set do not have to present the same sequence separation as the candidate structural motif defined in the motif set). Since the sequence separation between pieces is larger than all other sequence separations, this value is typically referred to as the long delta of a candidate structural motif and allows capturing long-range interactions. Given the notation above, candidate structural motifs are grouped based on the number of residues (*i.e.*, *M* and *N*) in each piece of residues comprising the candidate structural motif and are referred to as *M* + *N* motif candidates. In addition to the principal set of non-contiguous candidate structural motifs, where all residues within each piece, *P*_1_ and *P*_2_, had to be consistently classified as adopting either helix or strand conformation, we also created candidate structural motifs sets where one or both of the two pieces, *P*_1_ and *P*_2_, was allowed to adopt a coil secondary structure instead of having either a helix and/or strand secondary structure.

Since it is not verified that there is an interruption of the secondary structure element between *P*_1_ and *P*_2_, a structural motif can, in principle, be created from a single secondary structure element, a helix with a kink, for example. However, because of recurrence requirements (described in more detail below), only packings that reappear several times will be present in the results. Note also that the two pieces of secondary structures do not need to follow directly after each other; they can be far apart in the sequence and separated by other helices, strands or coil regions that do not take part in the recurrent candidate structural motif. Each of the two pieces *P*_1_ and *P*_2_ consist of two or three residues, that can themselves be separated by up to five amino acids and such that each piece is exclusively and entirely in a helix, a strand or a coil.

### 3.4. Structure Properties of Non-Contiguous Candidate Structural Motifs

As mentioned earlier, the residue separation(s) within each piece, which we refer to as “short delta(s)”, are forced to remain constant in the searches, while the residue separation between two pieces, which we refer to as “long delta”, are allowed to vary. Such long deltas are allowed to deviate from their original value by ±100, but may, in doing so, never be less than six. Pieces must appear in the same sequential order as in the corresponding structural motif. For each of the residues in one piece, there should be at least one so-called non-local contact to the other piece, where contacts between residues are considered to exist whenever a shorter than 10 Å edge is present in a corresponding Delaunay triangulation [50,51] of the atom coordinates of the protein structure in question. For this purpose, Delaunay triangulations were calculated for point-sets containing the coordinates of C^α^-atoms only and for point-sets containing the coordinates of all heavy atoms in each PDB-file. The following combinations of candidate structural motifs were created: 2 + 2, 2 + 3, 3 + 3 and 2 + 4. Other motif definitions will be investigated in future studies. All possible C^α^–C^α^, N^H^–N^H^, C^O^–C^O^, N^H^–C^α^, C^O^–C^α^, N^H^–C^O^ and O–N^H^ distances between residues were used to specify the conformation of each candidate structural motif, so that only constraints involving the backbone are used in this first step. During the search to retrieve comparable arrangements of residues in other structures, distance tolerances were set to ±20% of the original distance (minimum 2.0 Å, maximum 4.0 Å).

### 3.5. Identification of Recurrent Non-Contiguous Structural Motifs

For all non-contiguous RSM candidates satisfying these coarse distance constraints, the RMSD is automatically calculated; therefore, the exact value of the distance tolerance is not critical. The main purpose of the distance tolerance is to discard hits as soon as one distance deviates significantly from the corresponding motif in the candidate structural motif to save time and excessive printouts of results for meaningless hits. The combination of distance tolerances and RMSD cutoff ensures that the obtained RSMs are well defined with respect to the backbone. The type of amino acid has not been allowed to change in the searches presented in this paper (but, it is possible to do so for SwissPDBViewer searches). Although candidate structural motifs are created from pieces of exclusively helix, strand or coil conformation (to keep the number of candidate structural motifs within an acceptable range), the secondary structure is allowed to vary freely in the searches (as long as the distance constraints are not violated). All proteins that have a chain related to the one used to define the candidate motif (BLAST *E*-values less than 10, according to tables downloaded from RCSB protein data bank (www.rscb.org)) were omitted in the searches, so as to collect only hits from proteins unrelated to the protein used to define the candidate structural motif. In addition, a subsequent filtering is done after the searches, so that for each candidate structural motif, only hits coming from mutually unrelated proteins are kept when calculating the recurrence level of a motif. This is done so as to simulate a situation where no homology modeling templates are available or where the templates that can be identified are so distant that it is difficult to build a high confidence model. In other words, we want to exclude similarities due to common origin and focus on favorable ways of packing non-contiguous residues. Since we want to have as many reusable RSMs as possible, we prefer searching in a large database (99%) and do subsequent filtering instead of reducing the number of proteins beforehand.

The recurrence level of a RSM is calculated by ordering supporting chains in decreasing order by how many times each chain supports the RSM, eliminating every chain that is related to any previously occurring in an un-eliminated chain and then counting all chains that remain.

### 3.6. Computational Alanine Scanning

Computational alanine scanning calculations were performed using the empirical force field, FoldX (version 6.0) [52,53]. For three protein chains (1o75-A, 1s9r-A and 3jzm-A) out of the 5,489-element motif set, no results could be obtained, and these are excluded from the results for the sections that report amino acid compositions and folding free energy changes. Aside from the three excluded chains, calculations were performed for all standard amino acid residues of all chains in the motif set.

## 4. Conclusions

We have presented a large collection of non-contiguous structural motifs that have reoccurred in multiple unrelated protein structures. The repeated occurrences of these motifs in unrelated structures suggests that they are intrinsically stable, as supported by the *in silico* alanine mutations results, and that they represent nearly ideal spatial packing patterns. By their nature, these motifs thus correspond to statistically more likely geometrical arrangements of sequentially non-contiguous amino acid residues and may therefore be of value for inter-residue contact prediction and protein structure refinement and, more particularly, for the refinement of structures using sparse and/or low-resolution data. Detection and identification of structural similarities, as shown in Section 2, is a concrete area in which these recurrent structural motifs have demonstrated their usefulness. We also envision that the design of more stable proteins could be facilitated by the utilization of the recurrent structural motifs presented here.

## Supplementary Information



## Figures and Tables

**Table 1:** Summary of recurrent structural motifs (RSMs) with backbone similarity requirements only. Summary of the number of recurrent non-contiguous structural motifs of different types and the following slash-symbols: the average degrees of recurrence, for different backbone root mean square distance (RMSD) cutoff levels (1 to 5) in increasing order from top to bottom with a step size between rows of 0.1 Å. The cutoff values are 0.4 to 0.8 Å for 2 + 2 RSMs, 0.5 to 0.9 Å for 2 + 3 RSMs and 0.6 to 1.0 Å for 3 + 3 and 2 + 4 RSMs. In parentheses: corresponding lower bounds on the number of distinct recurrent non-contiguous structural motifs (see end of Section 2.2 for details).

	Level	2 + 2 RSM (0.4–0.8 Å)	2 + 3 RSM (0.5–0.9 Å)	3 + 3 RSM (0.6–1.0 Å)	2 + 4 RSM (0.6–1.0 Å)
RSMs entirely composed of secondary structure elements	1	406,720 (127,807)/5.4	103,711 (68,911)/3.0	5,612 (4,802)/2.4	3,896 (3,362)/2.4
	2	648,493 (210,897)/5.6	156,654 (104,859)/3.0	7,202 (6,149)/2.4	4,977 (4,299)/2.4
	3	890,876 (298,048)/5.7	211,128 (142,866)/3.0	8,487 (7,229)/2.4	5,958 (5,161)/2.4
	4	1,136,125 (389,668)/5.8	261,410 (178,977)/2.9	9,491 (8,095)/2.4	6,678 (5,792)/2.4
	5	1,364,736 (480,393)/5.8	303,198 (209,707)/2.9	10,245 (8,773)/2.4	7,206 (6,258)/2.3

RSMs containing coil regions	1	8,931 (6,766)/4.8	2,304 (1,978)/3.0	155 (138)/2.4	143 (132)/2.3
	2	14,602 (11,599)/4.9	3,169 (2,773)/3.0	189 (171)/2.4	168 (157)/2.3
	3	22,178 (18,236)/4.9	4,064 (3,622)/3.0	209 (191)/2.4	198 (186)/2.3
	4	32,815 (27,539)/4.8	4,922 (4,446)/3.0	222 (204)/2.4	210 (198)/2.3
	5	46,953 (39,806)/4.6	5,702 (5,196)/3.0	240 (222)/2.4	217 (205)/2.3

When calculating an RSM’s level of recurrence, the chain from which the motif has been generated is ignored. The total number of occurrences in the search set is, thus, at least one higher than the reported level of recurrency.

**Table 2:** Summary of RSMs with similarity requirements for all heavy atoms. Summary of the number of recurrent non-contiguous structural motifs of different types and following slash-symbols: the average degrees of recurrence, for different RMSD cutoff values, for all heavy atoms, in increasing order from top to bottom with a step size between rows of 0.1 Å.

	2 + 2 RSM (0.4–0.8 Å)	2 + 3 RSM (0.5–0.9 Å)
RSMs entirely composed of secondary structure elements	21,452/4.2	7,133/2.9
	49,132/4.6	14,139/3.1
	92,774/4.9	24,247/3.1
	151,704/5.1	37,851/3.1
	229,068/5.2	56,275/3.1

RSMs containing coil regions	1,505/4.0	487/2.8
	2,655/4.4	780/2.8
	4,185/4.5	1,066/2.9
	6,028/4.6	1,437/2.9
	8,399/4.6	1,841/3.0
