# Supplementary material for: Recurrent Structural Motifs in Non-Homologous Protein Structures
Source: Int J Mol Sci. 2013 Apr 10;14(4):7795–814. doi: 10.3390/ijms14047795 (PMC3645717; doi:10.3390/ijms14047795)
Supplement: Supplementary file 1 [file ijms-14-07795-s001.pdf]

Supplementary Information

Table S1. Amino acid compositions in percent as seen over all residues in all chains (column 2), all candidate structural motifs (column 3), and all RSMs (column 4). Column 5 presents the amino acid composition when summed over all RSMs, counting each residue as many times as it is present in RSMs.

Amino acid	Protein chain	Candidate structural motif	RSM	RSM_{Σ}
V	7.0	7.2	14.4	25.5
I	5.8	6.0	10.8	15.3
L	9.6	9.8	14.4	17.5
P	4.5	4.4	1.7	0.8
Y	3.6	3.8	4.4	2.9
A	7.9	7.8	8.4	6.7
W	1.4	1.5	1.0	0.3
M	1.7	1.7	1.0	0.3
H	2.3	2.3	1.4	0.6
K	5.9	5.9	4.5	3.2
R	5.1	5.2	4.4	3.0
D	5.9	5.8	3.1	2.0
E	6.9	6.8	4.9	3.6
S	6.1	5.9	4.5	3.0
T	5.5	5.5	6.2	6.3
N	4.4	4.3	1.8	0.7
Q	3.9	3.9	2.2	0.9
C	1.3	1.3	1.0	0.4
G	6.8	6.5	4.6	2.9
P	4.5	4.4	1.7	0.8

© 2013 by the authors; licensee MDPI, Basel, Switzerland. This article is an open access article distributed under the terms and conditions of the Creative Commons Attribution license (http://creativecommons.org/licenses/by/3.0/).
